# Progressive edemas and generalized telangiectasia: A presentation of Intravascular B‐cell Lymphoma

**DOI:** 10.1002/ccr3.2514

**Published:** 2019-11-06

**Authors:** Johanna Vásquez, Vicente Romero, Pedro Vilas, José Antonio Serra-Rexach, María T. Vidán

**Affiliations:** ^1^ Department of Geriatrics Universitary Hospital Gregorio Marañón Madrid Spain; ^2^ Department of Dermatology Universitary Hospital Gregorio Marañón Madrid Spain; ^3^ Facultad de Medicina Universidad Complutense de Madrid Spain; ^4^ Instituto de Salud Carlos III CIBER of Frailty and Healthy Aging (CIBERFES) Madrid Spain

**Keywords:** edema, intravascular lymphoma, large B cell, telangiectasia

## Abstract

Intravascular B‐cell Lymphoma is a rare lymphoproliferative disorder with a none specific clinical presentation. The association of cutaneous telangiectasia‐like lesions and elevated inflammatory markers should be guaranteed a skin biopsy.

## Graphical abstract

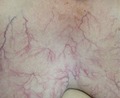

## 1 INTRODUCTION

Intravascular B‐cell Lymphoma (IVL) is a rare and aggressive subtype of extranodal non‐Hodgkin's lymphoma, characterized by the selective growth of lymphoma cells within lumen of capillaries and small vessels. IVL was described first by Pfleger and Tappeiner in 1959 as a systemic proliferating angioendotheliomatosis.^1^

About 40% of IVL presents skin lesions, most common cutaneous manifestation is subcutaneous nodules and plaques. The clinical presentation is highly variable. Generalized edema and telangiectasia have been occasionally described in the literature.^2^

We present a case of IVL with initial cutaneous involvement consisting in telangiectasias and anasarca.

## 2 CASE REPORT

A 91‐year‐old woman was admitted to the acute care geriatric unit presenting progressive edemas, asthenia, and functional impairment over 2 months. Her medical history included Parkinson disease, mild cognitive impairment, Polymyalgia rheumatica, and diverticulitis.

Previous treatment included Omeprazol 20 mg, L‐dopa/carvidopa 87.5/350 mg, Furosemide 20 mg, Quetiapine 50 mg, Rivastigmine 4.6 mg, and Alopurinol 100 mg. The woman was partially dependent on basic activities of daily living (Barthel index 60/100). She required a frame and the assistance of another person to walk. (Functional Assessment Classification 2).

**Figure 1:**
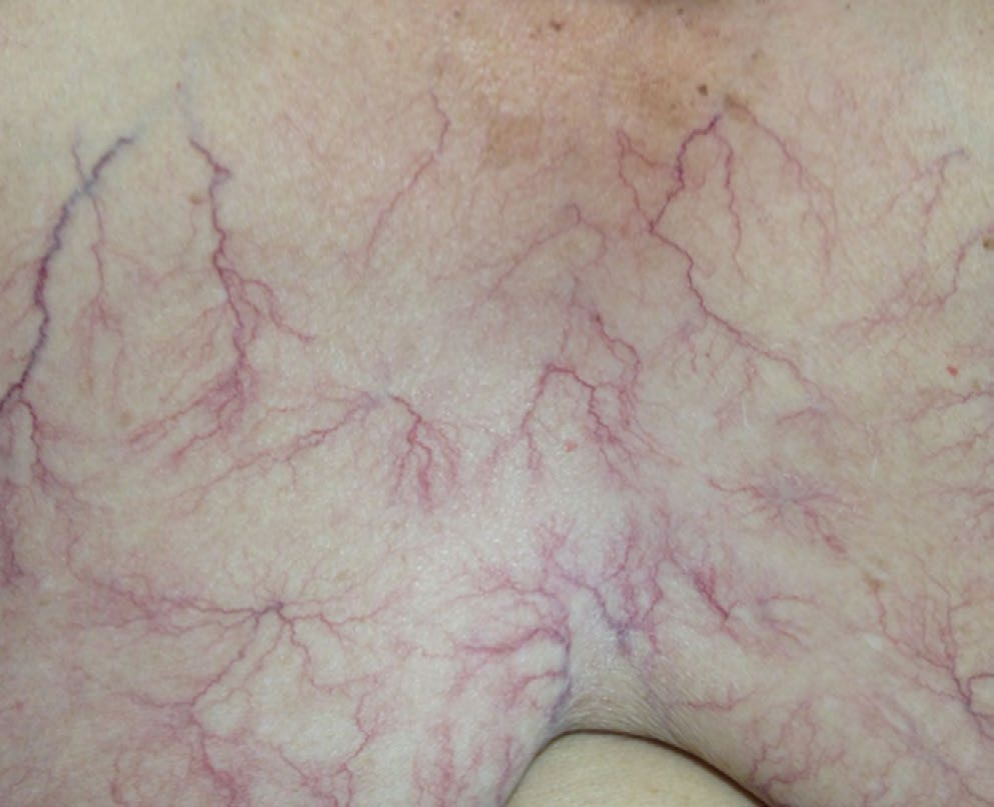
Generalized telangiectasia in chest

Physical examination revealed severe edemas in legs, abdomen, and breasts. Furthermore, vascular lesions resembling telangiectasia in chest (Figure 1), back, and abdominal regions were observed. Her heart rate and rhythm were regular. Lungs auscultation was normal. No ascites signs were found in the abdominal examination.

Laboratory findings were as follows: normocytic anemia (red blood cell count 3.00 × 10^12^/L, hemoglobin level 9.8 g/dL, mean corpuscular volume 92 fL), elevated serum levels of lactate dehydrogenase (969 mg/dL), and serum ferritine (892 mg/dL); NT‐proBNP was 1800. Total serum protein was 6.2 g/dL (normal range 5.5‐9.0 g/dL) with an albumin in normal levels. Tumoral markers were negative. Serology for Epstein‐Barr virus, citomegalovirus, Rickettsia, and Leishmania were negative.

Chest X‐ray was normal. Body CT (computed tomography) scan showed generalized edema in the lateral abdominal wall, pelvis, and inferior extremities at the subcutaneous cellular tissue level. Deep venous system was permeable. No findings suggesting solid lesions. No pleural, pericardial, and peritoneal effusions were found. The transthoracic echocardiogram (TTE) and breast ultrasound showed no findings. Other possible causes like cardiopathy, liver disease, renal impairment, malnutrition, rheumatologic conditions, or pharmacologic toxicity were excluded.

Diuretic treatment was started, and mild improvement of edema was observed with objective weight loss about 10 kg.

**Figure 2:**
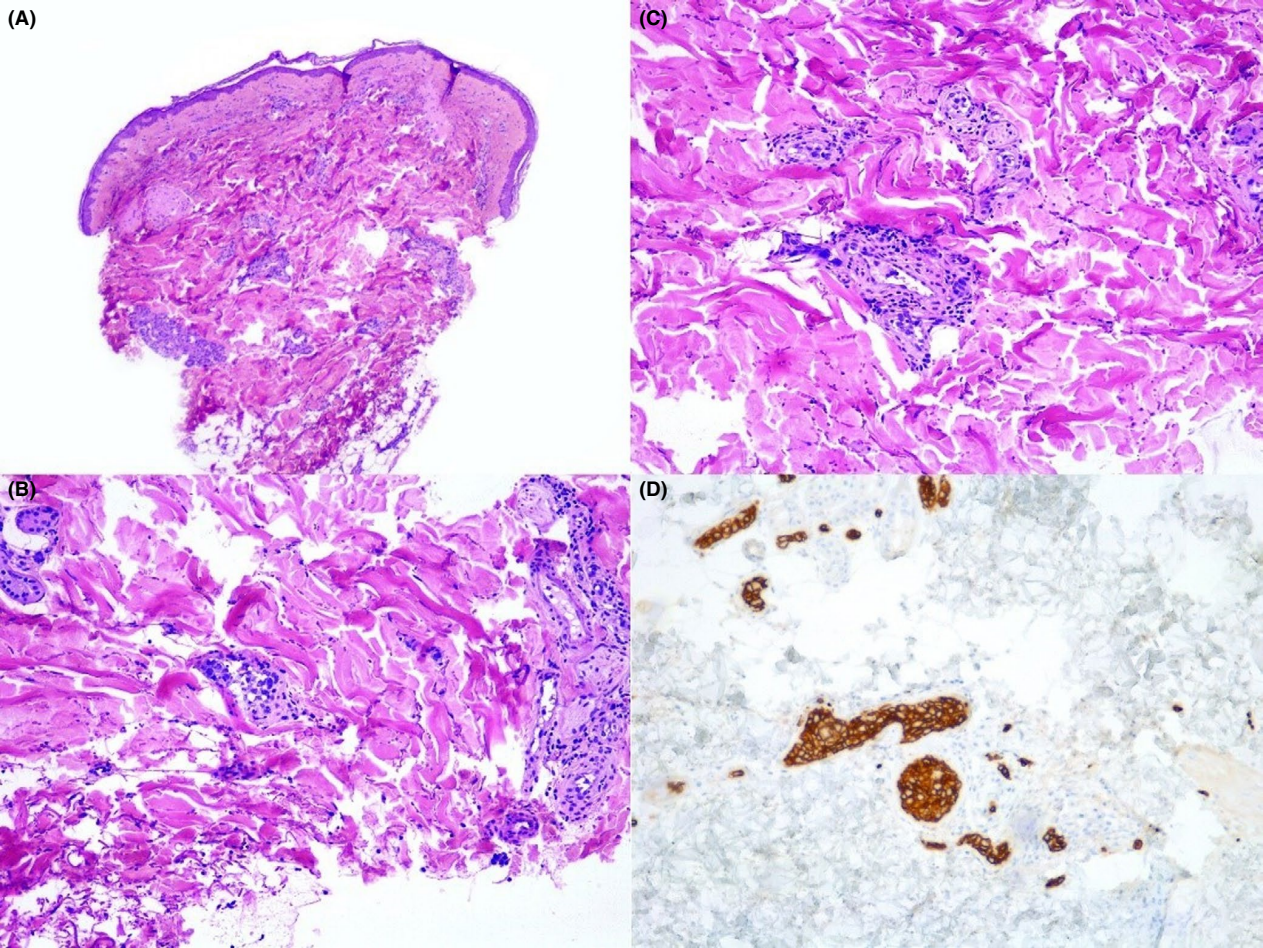
A, H&E ×40 Dilated vessels in the dermis (B) and (C) H&E ×400, dilated vessels in the dermis with a reactive perivascular infiltrate, detail of the large atypical lymphocytes within the vessels. D, Neoplastic cells are positive for CD20

Due to the persistence of asthenia, functional compromise, telangiectasia, and no clear diagnosis at that moment, a biopsy of the skin lesion was made. The histopathological analysis revealed a proliferation of large lymphocytes filling dilated blood vessels within the dermis and subcutaneous tissues; the neoplastic cells were large with scant cytoplasm and a prominent nucleoli. These lymphocytes were positive for CD20, CD79a, confirming their B‐cell nature (Figure 2). With the confirmed diagnosis of IVL, a hematologist was consulted. We decided to offer our patient conservative treatment due to progressive functional deterioration, short‐term poor prognosis and considering that the risks of treatment outweighed the benefits.

At discharge, the patient had almost complete resolution of anasarca but persistent breast edema without modification of skin lesions. Laboratory findings demonstrated improvement of cholestasis and acute phase reactants. The LDH remained elevated (865 U/L). As a consequence of edema reduction, she partially recovered her ability to walk.

The following days, during the stay at home, her evolution was unfavorable. She had several fall episodes whit humerus fracture and progressive functional impairment. After 45 days from discharge, the patient had a readmission due to her critical status in relationship to a sepsis secondary to a perforation from colonic diverticulitis. She died 24 hours after the readmission.

## 3 DISCUSSION

IVL is an uncommon subtype of extranodal non‐Hodgkin's lymphoma. The incidence is estimated in <1 million for a person, with a middle age about 70 years and no difference between sex.^3^, ^4^

It is characterized by the selective growth of lymphoma cells within lumen of capillaries and small vessels. The absence of crucial molecules for adhesion of lymphocytes to vascular endothelium can play a critical role in its development.^5^

The clinical presentation is none specific, ranging from monosymptomatic forms, such as fever, pain, or local symptoms, to the combination of B systemic symptoms (fever, night sweats, weight loss) and rapidly progressing manifestations of multiorgan failure due to occlusion of small vessels in different organ systems.^3^ There are two types of clinical presentations across different countries. In western regions is more common the presence of symptoms related to compromise of the central nervous system (CNS) and skin. Asian countries frequently present with affection of the bone marrow, spleen, and liver.^6^

About 40% of IVL presents skin lesions, typically tender indurated erythematous to purple plaques and nodules, often distributed over the trunk and extremities, accompanied by circumscribed edema in some cases. Because of the variation in cutaneous findings, the IVL differential diagnosis is quite broad.^7^ These skin manifestations often are misdiagnosed as cellulitis, erythema nodosum, vasculitis, skin cancer, Kaposi sarcoma, or panniculitis.^8^, ^9^

Superficial telangiectasias overlying the indurated areas have occasionally been described.^2^, ^10^ In our case, telangiectasias associated with the presence of generalized edema were striking. Initially, we attributed these symptoms to an unknown systemic cause. Because of an elevation of inflammatory markers, many diagnostic tests were made with no results for infectious or autoimmune etiology, neither for heart failure. A solid neoplastic cause was delayed by extension studies including breast ultrasound and Body CT scan.

The skin biopsy has an important role in the diagnosis, even in cases without cutaneous involvement. There are reports describing the random skin biopsy of healthy‐appearing skin as a tool of remarkable diagnostic value for IVL.^8^ In our patient, the level of suspicion was low because congestive symptoms, like anasarca, were taken into consideration, thus shadowing our initial diagnosis. In the end, skin biopsy, including inmunohistochemical tests, confirmed the diagnosis.

IVL usually has a fulminant natural history with a rapid evolution. However, the median survival is approximately 5 years in the cases with early diagnosis. In some series, it has been described that the cutaneous variant of the classic subtype has better prognosis.^3^, ^6^, ^11^

As treatment, a combination of cyclophosphamide, doxorubicin, vincristine, and prednisone with the recombinant anti‐CD20 antibody rituximab (R‐CHOP) is the most common treatment and may achieve complete remission and improve clinical outcome with 24‐month survival rate of 66% in the largest retrospective study.^12^

It has also been suggested that adding drugs with a higher bioavailability in the central nervous system (CNS), such as methotrexate or cytarabine may be helpful, mainly in patients whit such affections. Autologous peripheral blood stem cell transplantation has been used occasionally in patients with promising results, but data on large numbers of patients are lacking.^5^

## 4 CONCLUSION

Our case was an unusual presentation of IVL. The extreme difficulty in diagnosis was related to the confounding myriad of clinical manifestations. It is important for the clinicians to be alert about this type of disease, reflecting on its association with generalized edemas and telangiectasia. Furthermore, in older patients who present cutaneous telangiectasia‐like lesions and elevated inflammatory markers, a skin biopsy should be guaranteed. This test is an easy, accessible, and minimally invasive method that may allow an early diagnosis and appropriate treatment, hence improving patient survivability.

## CONFLICT OF INTEREST

The authors declare no conflict of interest.

## AUTHOR CONTRIBUTIONS

JV: wrote the first draft, performed the data collection from clinical history, and completed an extensive literature search. MV: was the attending geriatrician in charge of the case. JV and VR: participated in the acute and ongoing medical care of the patient. JV, VR, MV, and JS: polished the article, added to the review of the literature, and were involved in the ongoing medical care as well the complications presented afterward. PV: was the dermatologist in charge of the case and was responsible for the histological photographs and descriptions and provided images.
